# Attention and distraction in the predictive brain

**DOI:** 10.1080/13506285.2021.1936733

**Published:** 2021-09-28

**Authors:** Heleen A. Slagter, Dirk van Moorselaar

**Affiliations:** a Department of Experimental and Applied Psychology, Vrije Universiteit Amsterdam, Amsterdam, The Netherlands; b Institute for Brain and Behavior, Vrije Universiteit Amsterdam, Amsterdam, The Netherlands

**Keywords:** Attentional capture, statistical learning, predictive processing, inhibition, visual search

## Abstract

Whether it is possible to ignore a physically salient distractor has been a topic of active debate over the past 25 years, with empirical evidence for and against each of the theoretical stances. We put forward that predictive processing may provide a unified theoretical perspective that can account reasonably well for the empirical literature on attentional capture. In this perspective, capture is a logical consequence of the overall imperative of the brain to predict what sensory signals provide precise information to achieve goal-directed behaviour.

We applaud Luck et al. (2021) for their comprehensive historical overview of the attentional capture debate and for delineating the current theoretical consensus and remaining disagreement. Such adversarial discussion is key to scientific progress and ultimately, for explaining the nature of distraction. We contend that in moving the debate forward it is also critical to consider *why* each of the opposing theoretical stances is supported by data. We posit that an essential key towards answering this question lies in understanding attentional capture from the perspective of the predictive brain (Friston, 2010). This perspective explains how selectivity naturally emerges in a system that needs to adaptively interact with its environment, that must select one action over the other, and that to do so, must learn to predict which aspects of the environment are overall informative. Within this perspective, attentional capture is an ineluctable consequence of prediction, and constructs like goal-directed attention, implicit learning and physical saliency (i.e., the three main selection influences identified in Luck et al., 2021) are logically united by the continuous drive of the brain to reduce long-term prediction errors associated with sensory exchanges (Friston, 2009). First, we discuss the predictive processing perspective and review how an appeal to predictive processing may allow for an integrative understanding of the attentional capture literature and reinterpretation of recent findings. We then briefly review recent neuroscientific findings that shed light on how predictions may reduce distractor interference. In doing so, we suggest important avenues for future research.

The dominant view in cognitive neuroscience, taught in the vast majority of text books, is that the brain, like a computer, *processes* information in stages: from sensory encoding to decision making to action selection. This is also how the brain is typically empirically studied: participants are presented with stimuli to which they have to respond and the externally induced neural activity is correlated to their behavioural responses. Yet, in recent years, it is increasingly recognized that because the brain has no direct access to the external world, only to the incoming signals conveyed by the senses (i.e., its *own* activity patterns), in order to adaptively interact with its environment, the brain must instantiate predictive models about the hidden causes of its sensory input (Friston, 2010). Moreover, to construct reliable models, the brain must rely on action, as it is only through action that the brain can, like a scientist, manipulate the incoming signals and determine whether the action-induced changes in its sensory activity align with its model’s predictions of what is out there. By, for example, taking a closer look, the brain can verify the accuracy of its predictions. In this view, the brain is thus in charge of *generating* information, to give sensations meaning and guide actions, and continuously aims to minimize prediction errors to ensure model fitness. Plasticity is emphasized, as the brain constantly adapts based on changes in input structure. Yet, the aim is not to create a mirror-like copy of the outside world per se, if at all, but to best predict action-induced sensory outcomes across multiple time scales, so that the larger system that the brain is intrinsically part of (i.e., the body), can sustain itself as a living organism (Allen & Friston, 2018). Brains are ultimately about organizing action (Hommel et al., 2019). Indeed, a growing body of research shows that even in early sensory brain regions, responses also reflect predictions (Kok et al., 2013) and actions (Schneider, 2020).

In a brain engaged in predictive processing, unexpected external events will generate large prediction errors, that it needs to explain. Yet, it would be detrimental if predictive models would simply be at the whim of external influences, as the world is full of statistical noise or uncertainty. The brain hence also has to deal with statistical volatility and predict the precision (inverse variance) of sensory evidence (prediction errors) (Friston, 2009). Precision weighting ensures that only prediction errors with high expected precision, that provide high-quality sensory information, can revise predictive models. This provides the brain with a mechanism to control the relative influence of top-down predictions vs. bottom-up input in a context-sensitive manner. It has been proposed that attention maps onto precision weighting, in line with empirical findings that attention can modulate sensory gain (Feldman & Friston, 2010; Hohwy, 2012).

Although it is far from clear if predictive processing can account for all mental phenomena, this framework may provide a unified explanation for seminal findings in the attentional capture literature, as the three main selection factors (physical salience, implicit learning, explicit goals) simply relate to the same mechanism: precision weighting. It can firstly explain why it is so difficult to ignore a physically salient distractor to begin with. Strong sensory input (e.g., driven by large spatial contrast and/or temporal contrast (abrupt onset) stimuli) is expected to have a better signal-to-noise ratio (higher precision) than weaker input (Feldman & Friston, 2010). Thus, high-contrast, physically salient stimuli are by default assigned high precision, rendering it more likely that they will capture attention. It may also explain why it is so difficult to overcome their capture. The expectation that strong input is precise is grounded in a life-time of learning across many different external environments, and thus relatively stubborn or insensitive to new experiences. Yet, in new task contexts with statistical structure, precision expectations can develop that restructure information sampling and reduce or even eliminate attentional capture. For example, the statistical structure of contexts in which the distractor is predictable across trials (Gaspelin & Luck, 2018) allows for the downweighting of distractor signals, as the brain learns that they convey low-quality information to the task at hand. Similarly, in feature search mode, when target features are predictable across trials, the precision of target signals can be upregulated, reducing the precision assigned to other stimuli. Yet, in statistically volatile contexts, as for example in mixed-feature variants of the additional singleton paradigm, in which targets and distractors randomly swap features across trials (singleton detection mode), precision expectations cannot develop, leaving physically salient stimuli the floor. Furthermore, the overarching nature of the expectation of the brain that strong sensory input is precise can explain why implicit distractor learning is task specific and does not transfer to novel tasks contexts (Britton & Anderson, 2020). Finally, predictive processing can also explain why a distractor is more likely to capture attention when it matches prioritized information (contingent involuntary orienting hypothesis (Folk et al., 1992)): its matched features are assigned higher precision.

Thus, within the predictive processing framework, the brain not only recapitulates the (statistical) structure of how sensations are caused, but simultaneously incorporates estimations of levels of uncertainty that can be adjusted on the basis of new learning. On the one hand, this can explain the seemingly involuntary nature of bottom-up, exogenous attention (overarching expectation that high-contrast stimuli are precise), as well as why the attractiveness of objects initially favoured by physical salience can reduce with new implicit learning (precision reweighting), as described above. On the other hand, it also explains why we *can* volitionally direct our attention, based on explicit goals (i.e., facilitated target processing in response to a cue), regardless of our previous experiences with a given visual environment or context. We have oriented our attention in space to many different objects in many different visual environments, and to an object in many different positions in many different contexts. These conditional independencies in the external world are mirrored in the functional anatomy of the brain (Friston & Buzsáki, 2016) and allow the brain to generate expectations of reliable information at a particular location in space regardless of what it is or the precise context, and to expect reliable object information regardless of its location and specific context. That is, the statistical structure incorporated by the brain of how sensations are caused and their estimated noise level (precision) can also abstract across specific contexts. This gives top-down attention its volitional character. Yet, in the external world, where and what are often conflated, such as when objects are more likely to occupy a particular location in space in a given visual setting (e.g., microwave in a kitchen). These context-related statistical dependencies are reflected in precision weighting, causing context-specific implicit biases in top-down attention. This account is in line with empirical findings that goal-driven attention is task general, but implicitly learned attention is not (Addleman et al., 2018).

Within the predictive processing framework, learning is hence a pervasive feature of attention, as optimizing precision expectations necessarily relies on integrating information over time, within and across contexts. This can explain both the seemingly voluntary nature of top-down attention and the apparent involuntary nature of bottom-up attention, as well as the effects of new statistical regularities on attentional orienting as these permit the development of precision expectations that restructure information sampling (i.e., attentional biases). In this unified account, attentional capture is an emergent property of prediction, an overgeneralization of an adaptive principle, and the various factors (salience, implicit learning and explicit goals) are naturally connected by the overall imperative of the brain to sample the most informative sensory signals.

We also appeal to predictive processing to inform the current debate, delineated in Luck et al. (2021), whether (1) only implicit biases or also explicit goals can induce proactive suppression, and (2) whether it is at all possible to overcome capture by a highly salient distractor. As to the first theoretical disagreement, Folk and Remington posit that explicit goals can proactively suppress distracting features, while the signal suppression theory and the stimulus-driven account argue that only learned (implicit) biases can prevent attentional capture based on studies showing that suppression is not possible when the distractor is cued on a trial-by-trial basis. Within the predictive processing framework, explicit goals can only indirectly prevent attentional capture when informative about the upcoming target (e.g., an explicit cue informing about the colour or location of the upcoming target), as in this scenario, target signals can be assigned higher precision than distracting input. This may explain observations that in many situations, stimuli do not grab attention when they do not contain target properties (Folk et al., 1992). By contrast, physically salient distractors are by default assigned high precision and overcoming this overgeneralized expectation takes time such that in the absence of precise target expectations, suppression of physically salient stimuli is dependent on implicit learning. The predictive processing account makes the additional prediction that benefits of implicit distractor learning are context- or task-specific, as also shown by recent studies (Britton & Anderson, 2020). To support their claim, Folk and Remington reference two studies, which in our view do not provide unequivocal evidence for explicit proactive suppression. In the first study by Lien et al. (2010), equal capture by distractors was found in blocks in which the target colour was fixed versus randomly cued from trial to trial. Thus, only target foreknowledge was manipulated, and their results hence cannot provide evidence for the notion of direct top-down suppression by explicit distractor foreknowledge. Although the study by Moher et al. (2011) showed reduced capture by colour singleton distractors after cues signalling a high distractor probability relative to low probability distractor cues, critically, response times were also slowed on distractor absent displays following high probability distractor cues. This strategic slowing of response times indicates that results reflect slow endogenous shifts of attention rather than the absence of exogenously driven attentional capture (Hickey et al., 2010). Moreover, participants were first trained with a version of the task in which the high and low distractor probability cues were not randomly intermixed within a block, permitting statistical learning that may have induced implicit biases in the subsequent task. To univocally show suppression by explicit goals, one needs to demonstrate suppression when the distractor is cued on a trial-by-trial basis in the absence of any possibility of prior implicit learning or information about the target stimulus.

While there is a general consensus that implicit learning shapes capture, a second theoretical disagreement concerns whether implicit learning-related proactive suppression of distractor features can be strong enough to overcome capture by *highly* physically salient distractors. Luck and Gaspelin summarize convincing evidence, from behavioural, eye tracking and ERP studies, that implicit learning can prevent attentional capture (Gaspelin & Luck, 2018). Yet, Theeuwes argues that capture cannot be overcome in all cases, largely based on a recent study by Wang and Theeuwes (2020). This study showed that while capture by a singleton distractor could be proactively suppressed in a small, set size 4 condition, replicating seminal work by Gaspelin and colleagues (Gaspelin et al., 2015), capture returned when set size was increased to six or ten items. Wang and Theeuwes reasoned that in the larger set size conditions, the singleton distractor was physically more salient, due to increased local feature contrast, and increased similarity to non-singleton distractors. Theeuwes therefore posits that proactive weighting of non-spatial features is possible, but cannot overcome high levels of physical salience. This is certainly plausible, given the stubbornness of the expectation that strong sensory signals generate reliable information. However, from the predictive processing perspective, it is also notable that the conditions differed in the extent to which distractor learning could occur. That is, in the small set size condition, the distractor could occur at fewer locations (4 vs. 6 or 10) and in fewer possible display configurations (72 vs. 900 or 56,700) than in the larger set size conditions. Thus, this condition was statistically much less variable. Moreover, only in the small set size condition, the distractor singleton had a unique shape, which likely also enhanced the ability to anticipate the distractor in this condition. Thus, in the larger set size conditions, the strength of the “attend-me” signal triggered by the singleton distractor may have been stronger, not simply because it was physically more salient, but also because of reduced familiarity with the distractor and its context. To unequivocally demonstrate that capture by a highly physically salient stimulus is inevitable, future studies will need to control for potential differences between conditions or experiments in overall distractor predictability and contextual learning. One testable prediction that derives from the above is that provided enough opportunity for learning, attentional capture will also disappear in the larger set size conditions. If observed, this would argue against the stimulus-driven account put forward by Theeuwes that capture by highly physically salient cannot be overcome. Indeed, other work shows that capture by highly physically salient, abrupt-onset stimuli can disappear (Turatto et al., 2018), already nuancing this claim.

The above illustrates the strong influence of implicit learning on capture, and the difficulty of examining the individual selection influences in isolation, indicative of their intertwined relation. It also raises the question how distractor expectations are neurally implemented. As visualized in Figure 2 in Luck et al. (2021), it is currently unclear how, if at all, the gain of non-spatial features can be modulated prior to saliency computations. Supporting the notion that the brain continuously predicts upcoming input, recent studies show that predictions tune sensory processing towards the expected stimulus, and this tuning becomes more precise when the expected feature is also attended (Walsh et al., 2020). However, it is currently highly controversial whether the brain also implements “templates for rejection,” and if so, whether such templates can be flexibly instantiated (through explicit goals) or only manifest itself as a function of implicit learning. While to date, in line with the behavioural literature, there is no neural evidence yet in support of flexible distractor templates (i.e., when the distractor is cued on trial-by-trial basis; de Vries et al., 2019; Reeder et al., 2018), we recently showed that with implicit learning both the expected spatial frequency of the target, and that of the distractor could be decoded from pre-stimulus brain activity (van Moorselaar et al., 2020). Strikingly, the classification algorithm did not generalize from distractors to targets, suggesting that while the brain continuously predicts upcoming input based on sensory regularities in the environment, target and distractor expectations are qualitatively different in nature. Although this warrants confirmation, learning what is relevant may induce feature upweighting, whereas distractor learning results in feature downweighting.

Figure 2 of Luck et al. (2021) also illustrates that all models agree that through implicit learning, likely distractor locations can be proactively suppressed. Yet to date, there is little evidence that spatial expectations are actually encoded in pre-stimulus neural tuning. Although one study observed proactive changes in distractor location representation, as reflected in pre-stimulus alpha-band asymmetry (Wang et al., 2019), four other EEG studies in which the distractor location became predictable across trials did not observe any anticipatory changes in the representation of this location (Noonan et al., 2016; van Moorselaar et al., 2020, 2021; van Moorselaar & Slagter, 2019). One possibility is that distractor location learning is implemented via an activity-silent mechanism or synaptic plasticity, and only becomes apparent once distractor foreknowledge can be integrated with bottom-up sensory input. Indeed, distractor location learning has been associated with modulations of the Pd, an event-related potential linked to distractor inhibition. Thus, feature and spatial distractor expectations may be differentially implemented in the brain as a function of implicit learning. Future work is necessary to further understanding of how the brain learns to expect (ignore) irrelevant, distracting information.

To conclude, we put forward that predictive processing may provide a unified theoretical perspective that can account considerably well for the empirical literature on attentional capture. In this perspective, capture is the logical consequence of the overall imperative of the brain to predict what sensory signals provide precise information to achieve goal-directed behaviour. There is no homunculus that directs attention or inhibits distractors, but simply a brain that attempts to incorporate the structure of the world in a reliable and meaningful way.
